# Design and validation of the CADIS-G: a screening instrument for anxiety, depression, loneliness and anger in the older population

**DOI:** 10.3389/fpsyt.2025.1591296

**Published:** 2025-05-13

**Authors:** Elena Ruiz-Sancho, Francisco J. Román, Lucia Torices, Francisco Sánchez-Escamilla, Andrea Aguirre, Enrique Rubio, Patricia Cañada, Natalia Rodríguez, Leticia León, Miguel Ángel Pérez-Nieto, Marta Redondo-Delgado

**Affiliations:** ^1^ HM Faculty of Health Sciences, Universidad Camilo José Cela, Madrid, Spain; ^2^ HM Hospitals Health Research Institute, Madrid, Spain; ^3^ Department of Biological and Health Psychology, Universidad Autónoma de Madrid, Madrid, Spain; ^4^ Institute of Emotion and Health Psychology, Madrid, Spain; ^5^ Saturno Labs, Madrid, Spain

**Keywords:** anxiety, anger, depression, loneliness, older adults, screening scale

## Abstract

**Introduction:**

The assessment of psychological variables in older adults is often conducted using instruments that are not specifically designed for this population. Moreover, there are few screening tools that are effective for healthcare professionals in detecting emotional difficulties in older individuals. Therefore, the objective of this study was to design a questionnaire that can be used as a screening instrument to measure emotional distress in older individuals. A 12-item instrument (CADIS-G) with a 4-point Likert-type response scale was developed and tested for validity.

**Method:**

186 older adults from eight residences and/or centers for older individuals in Spain. The participants completed, in addition to the 12-item CADIS-G, different instruments considered gold standards: Yesavage Geriatric Depression Scale (GDS-15), Geriatric Anxiety Inventory (GAI), State-Trait Anger Expression Inventory (STAXI-2) and UCLA Loneliness Scale. Exploratory factor analysis (EFA) was performed to study the dimensionality of the CADIS-G items.

**Results:**

The results showed that 10 of the 12 items of the CADIS-G grouped well with the theoretically proposed gold standard scales.

**Discussion:**

The CADIS-G is a screening instrument that shows adequate psychometric properties to measure anxiety, depression, anger and loneliness in older people.

## 1 Introduction

The aging of the global population is an undeniable reality. According to World Bank data, in 2022, people 65 years of age or older represented 9.8% of the world’s population. This percentage has doubled over the past six decades and is projected to increase to nearly 18% by 2050 (1). These statistics underscore the importance of addressing the needs of the older population, aiming to enhance their quality of life and the factors associated with it (2).

Depression is one of the strongest predictors of poorer quality of life in older adults (3). Old age is a phase of life in which there is a greater likelihood of psychosocial disorders such as grief, social isolation, physical disability, and cognitive impairment, all of which contribute to depression (4, 5). According to the World Health Organization (6), the prevalence of depressive disorder affects approximately 7% of individuals over 60 years of age, and depressive symptoms vary from 4.5% to 37.4%, depending on the assessment instrument used (7, 8). Anxiety disorders are also highly prevalent in this population (9, 10). Studies indicate that anxiety and depression are sometimes underdiagnosed, highlighting the need for appropriate instruments to improve detection and treatment (11).

Another dimension that affects the quality of life of older adults is social support. Data indicate that loneliness and social isolation are determining factors that exert negative effects on the physical and mental health of older individuals (12). One-third of this population experiences loneliness (13) and many health professionals have expressed increasing concern about this perception among older individuals (14).

Anger has been much less investigated in the elderly population (15). The studies reviewed provide data that are not entirely consistent. Some conclude that older adults experience and express anger less intensely than younger adults, that the frequency of situations generating anger is lower for older adults than for young ones (16), and that passive regulation strategies for anger are used more frequently by older adults (17). Other studies, however, conclude that older individuals report higher levels of cognitive aspects of hostility, including a greater number of hostile beliefs (18). The lack of studies highlights the need to explore in depth how anger affects the older population, as well as to develop instruments for evaluating anger in this demographic.

Despite the importance of depression and anxiety disorders, loneliness, and anger in the quality of life of older adults, these issues are often less addressed compared to those affecting other population groups (19, 20). This is, in part, due to the lack of instruments that allow for the simple detection of these issues and the saturation of the primary care system. Some studies have identified that older adults visit psychiatrists much less frequently than primary care centers (21); however, doctors at these centers fail to diagnose 40 to 50% of patients who suffer from various common mental disorders; (10, 22). Studies indicate that physicians focus more on evaluating physical illnesses, while patients demand greater attention to psychological and social problems (23). This fact highlights the need for primary care professionals to have screening tools for psychological aspects that affect the quality of life of older individuals. These tools should be simple and effective, thus improving the detection rate of these problems in patients aged 65 years and older (24).

To date, instruments designed for the general population are frequently used for such evaluations, without addressing the specific needs of the older population (25, 26). This implies that difficulties in distinguishing between cognitive and affective impairment are not taken into account, which makes it difficult to differentiate the somatic symptomatology of emotional problems from those found in other pathological processes, and the potencial effects of medication are not adequately addressed (27). Older individuals may present some of the symptoms that are being measured (28–30). There are some scales that measure depression and anxiety in older individuals, such as the Yesavage Geriatric Depression Scale (GDS) and the Geriatric Anxiety Inventory (GAI), which also have abbreviated versions; however, the number of items used varies depending on the study. There are discrepancies in results depending on the sex of the subjects, and the scales have been validated in a specific patient profile (31–33). Pocklington et al. (34) highlighted the difficulties in conducting a meta-analysis of various versions of the GDS due to the lack of standardization of the different abbreviated versions of the instrument. Therefore, continued research of the use of abbreviated versions of the GDS is necessary, along with the rationalization their use (35). Regarding the abbreviated versions of the GAI, reviews indicate that there are fewer studies on this subject and that more attention should be paid to the detection of anxiety disorders in this population (36). In relation to loneliness, few instruments have been specifically designed to assess it in older adults, with the UCLA Loneliness Scale being among the most widely used (37). However, further research is needed to assess loneliness more comprehensively in this population. Additionally, no instruments have been identified in the literature that specifically screen for anger in older adults. One example of available resources is the NSW Aged Health Network, which offers access to over 55 screening tools designed for older populations, addressing a variety of behavioral and psychological symptoms. Nevertheless, none of these tools assess anger. In terms of instruments that measure general distress, one notable example is the Kessler Psychological Distress Scale 10 (K-10), which has been validated in older adults across several countries (e.g., 38, 39) and more recently in Spain (40). While this instrument is effective in detecting distress, it primarily focuses on depressive and anxiety symptoms. The objective of this study is to broaden the detection of additional emotional challenges in this population, including anger and loneliness, which are also critical to their overall well-being. Typically, rapid screening instruments used in primary care for this population focus on identifying physical difficulties, such as vision or hearing impairments, or evaluating cognitive impairment and dementia (e.g., the Mini-Mental Status Examination [MMSE]) (41, 42). Therefore, we developed and tested the validity of a psychological screening instrument that allows a brief evaluation of anxiety, depression, anger and loneliness in older adults. The implementation of screening or detection tools appropriate to the healthcare reality is necessary because the early and simple detection of psychological problems will allow professionals to intervene in the most appropriate way or refer individuals for specialized mental health care when necessary.

## 2 Materials and methods

### 2.1 Participants

A total of 198 people (139 women [70.2%]; age: M = 79.14 and SD = 9.23) participated in the study between July 2022 and January 2023. Participants were recruited in eight nursing homes in Madrid, Segovia and Galicia as well as through the snowball method (47 people; 23.7%). All participants provided written informed consent to participate in the study. The final sample consisted of 186 individuals (130 women [69.5%]; age: M = 78.90 and SD = 9.21) after excluding individuals with more than 2 errors on the Short Portable Mental State Questionnaire (SPMSQ) (43; adaptation of 44). The inclusion criterion for participation in the study was being over 60 years of age (in accordance with the WHO recommendations for the age at which an individual is considered an older adult; 45). The exclusion criterion was cognitive impairment.

### 2.2 Procedure

The individuals who participated in this study completed several questionnaires to measure various sociodemographic and psychological variables (anxiety, depression, anger and loneliness). Sociodemographic variables were collected through an *ad hoc* questionnaire designed to gather the following information: sex, age, nationality, marital status, employment status, economic level, educational level, living status (alone or cohabitating) and other variables such as diseases, medication, sedentary lifestyle and physical activity. To measure the psychological variables, different questionnaires considered gold standards were used. The Yesavage Geriatric Depression Scale (GDS-15) (46; adaptation 47), the Geriatric Anxiety Inventory (GAI) (48; adaptation 49), the State-Trait anger expression inventory (STAXI-2) (50; Spanish adaptation by 51) and UCLA loneliness scale (52; University of California at Los Angeles) (53) were used. The participants then completed the CADIS-G (acronym in Spanish; translation, Anxiety-Depression-Anger-Loneliness Screening in the geriatric population) screening questionnaire to measure the previously mentioned psychological variables. The study was approved by the Ethics Committee of Camilo José Cela University (14_23_SPM, CEI-UCJC).

### 2.3 Instruments

#### 2.3.1 Geriatric Anxiety Inventory

The GAI is a 20-item questionnaire designed to assess anxiety in the older population. The items have a dichotomous response format, i.e., 1 is “agree” and 2 is “disagree”. For our study, taking into account that the directionality of all the items is positive, the responses were coded as 1 = “agree” and 0 = “disagree” so that higher scores reflect higher levels of anxiety. Studies have confirmed the adequate psychometric properties of the instrument (49, 54); however, there is disparity of opinions in relation to the factorial structure it presents. Some authors describe the instrument as one-dimensional (55), and others propose a three-factor structure (cognitive, activation and somatic) for the measurement of anxiety (56).

#### 2.3.2 Yesavage Geriatric Depression Scale (GDS-15)

The Geriatric Depression Scale was designed to evaluate depression in older people without overestimating somatic or neurovegetative symptoms (57). The authors developed a shortened 15-item version that has been widely used (58). The items have a Yes/No response format, with Items 1, 5, 7, 11 and 13 being inverse and being recoded to go in the same direction as the rest of the scale. For this study, “Yes” responses were coded as 1, and “No” responses were coded as 0. Thus, a higher score reflects a higher level of depression. The sensitivity and specificity values of the instrument are good, making it a widely recommended scale for the general screening of depression in geriatric patients (31, 59).

#### 2.3.3 State-Trait Anger Expression Inventory

The aim of STAXI-2 is to assess the different facets of anger. The Spanish version (51) has 49 items structured in 6 scales. For this work, a trait scale (10 items) was selected that measures how subjects perceive different situations as annoying or frustrating and the tendency to respond to these situations with anger. Although the other scales can yield interesting results, we prioritized the measurement of the general trait of anger for the screening. For this instrument, respondents score 10 items using a 4-point scale (1 = “almost never”, 2 = “sometimes”, 3 = “often” and 4 = “almost always”) based on how they feel normally. The authors reported adequate test-retest correlation values and good Cronbach’s alpha coefficients (51). Notably, we have not found any instrument that measures anger specifically in the older population; we decided to use the STAXI-2 because it has been applied in other studies of older adults (60, 61).

#### 2.3.4 UCLA Loneliness Scale

This scale is one of the most commonly used instruments to measure the feeling of loneliness. The original version has 20 items, but different shortened versions have been developed. The 10-item version developed by Russell in 1996 is used in this study (85). The items are scored from 1 = “I often feel that way” to 4 = “I never feel that way.” To favor the interpretation of the scale and to ensure that the responses are all in the same direction, the scores were inverted and coded as 1 = “I never feel that way” to 4 = “I often feel that way”. This scale has proven to be an adequate and sensitive instrument to measure the feeling of loneliness in older individuals and validated in this population (53).

#### 2.3.5 CADIS-G screening questionnaire

A screening questionnaire was designed for this study. The CADIS-G measures negative emotionality (anxiety, depression and anger) and the feeling of loneliness through 12 items. The response format is a Likert-type scale, with 0 indicating “Almost never”, 1 indicating “Sometimes”, 2 indicating “Often” and 3 indicating “Almost always”. To prepare the questionnaire, versions of items from different scales were selected and adapted to the aforementioned response format (with the exception of the item from the STAXI-2) to maintain internal coherence in the phrasing of the items on the screening questionnaire. In addition, some original items were added (see **Table 1**). Participants were asked to respond based on how they felt in the last week. The time needed to complete the questionnaire was approximately 5 minutes.

**Table 1:** Items on the screening instrument, measured variable and origin.

Item No.	Item (item direction)	Psychological variable measured	Origin
1	I have been feeling nervous (D)	Anxiety	GAI
2	I have spent a lot of time worrying (D)	Anxiety	GAI
3	I have had the feeling that my body is moving very fast (D)	Anxiety	Own elaboration
4	I have tried to avoid situations that cause me tension or anxiety (D)	Anxiety	Own elaboration
5	I have felt satisfied with my life (I)	Depression	GDS-15
6	I have given up many activities (D)	Depression	GDS-15
7	I have been in good spirits (I)	Depression	GDS-15
8	I have felt that my life is empty (D)	Depression	GDS-15
9	I have felt full of energy (I)	Depression	GDS-15
10	I have felt irritated (D)	Anger	STAXI-2
11	I have felt that I have no one to talk to (D)	Loneliness	UCLA
12	I have felt completely alone (D)	Loneliness	UCLA

GDS-15, Yesavage Geriatric Depression Scale; GAI, Geriatric Anxiety Inventory; STAXI-2, State-Trait Anger Expression Inventory; UCLA, UCLA Loneliness Scale; D, direct item; I, indirect item.

### 2.4 Statistical analysis

First, descriptive statistics were calculated for the sociodemographic variables (mean, standard deviation and frequencies). Second, the psychological variables considered gold standards (GDS-15, GAI, STAXI-2 and UCLA) were analyzed by calculating means, standard deviations, correlations between scales (Spearman) and the internal consistency (Cronbach’s alpha; α) of each scale. Third, the dimensionality of the CADIS-G instrument was analyzed. For this, exploratory factor analysis (EFA) was conducted, including the responses provided by the participants to the 12 items on the initial version of the CADIS-G, as well as the scores from the gold standard scales. The EFA was performed using generalized least squares (GLS) estimation and oblimin rotation. The suitability of the sample was evaluated by the Kaiser–Meyer–Olkin (KMO) test (62), and the strength of the relationship between the variables was assessed by the Bartlett test of sphericity (63). KMO values between 0.70 and 0.80 were considered good, values between 0.80 and 0.90 were considered excellent, and values greater than 0.90 were considered exceptional (64). Additionally, a significant Bartlett test result (p<0.05) was required to perform the EFA (65). Descriptive, correlation and reliability statistics were calculated for the final items of the CADIS-G. We evaluated the ability of the CADIS-G to distinguish between individuals with high levels of anxiety, depression, anger and loneliness using the cutoff points for the gold-standard scales [GAI (anxiety), GDS-15 (depression), STAXI-2 (anger) and UCLA (loneliness)] as criteria. Sensitivity, specificity, the Youden index, and area under the ROC curve were calculated for this purpose. The cutoff points used for each gold standard scale are presented below. For the GAI, a score greater than 10 has been established as a criterion. Values of 10/11 have been used previously, in other studies, as cutoff points for the original version (48) and the Spanish version (49). For the GDS-15, a score of 9/10 indicates symptoms of moderate depression. Regarding the STAXI-2, a score greater than or equal to 29 (the 90th percentile of the Spanish adaptation for the general population) was considered high. Finally, for the UCLA loneliness scale, a score greater than 30 (corresponding to a score of less than 20 on the original scale, where higher scores indicate higher levels of loneliness) was established as the cutoff point. This cutoff point has been used previously as an indication of a high degree of loneliness (53).

All statistical tests were considered bilateral, and p<0.05 was considered significant. The data were analyzed using the statistical program SPSS 26.0.

## 3 Results

### 3.1 Sociodemographic variables

This section provides the results of the descriptive analyses of the sociodemographic and health variables collected to characterize the sample. **Table 2** shows the frequency and percentage for categorical variables and the mean and standard deviation for continuous variables.

**Table 2:** Descriptive results for the sociodemographic and health variables.

	*N*	*%*	*M*	*SD*
Age			78.90	9.21
Sex (% women)	130	69.5		
Educational level				
No education	29	15.5		
Basic (EGB)	72	38.5		
Middle (FP)	41	21.9		
University	44	23.5		
Economic level				
Low	31	16.6		
Lower-Middle	34	18.2		
Middle	92	49.2		
Upper-Middle	24	12.8		
Upper	1	0.5		
Labor Situation				
Active	2	1.1		
Home worker	19	10.2		
Leave	2	1.1		
Retired	162	86.6		
Marital Status				
Single	26	13.9		
Married/In a relationship	82	43.9		
Separated/Divorced	14	7.5		
Widower	65	34.8		
Cohabitation				
Alone	64	34.2		
Partner	72	38.5		
Children	8	4.3		
Other relatives	6	3.2		
Other	37	19.8		
Medical Disease (% Yes)	157	84.0		
Diabetes (% Yes)	29	15.5		
Hypertension (% Yes)	96	51.3		
Rheumatological (% Yes)	59	31.6		
Cancer (% Yes)	22	11.8		
Respiratory (% Yes)	25	13.4		
Neurological (% Yes)	21	11.2		
Dermatological (% Yes)	12	6.4		
Others (% Yes)	31	16.6		
Psychological (% Yes)	51	27.3		
Medication (% Yes)	97	51.9		
Sedentary Life			4.12	3.20
Active Life			6.67	2.64
Regular Physical Exercise (% Yes)	144	77.0		
Exercise Type				
Aerobic	69	36.9		
Anaerobic/Other	38	20.3		
Aerobic and anaerobic	34	18.2		
Times per Week, Exercise			4.50	2.01

Most of the study participants had a basic education (38.5%) and were retired (86.6%). In terms of economic situation, more than 60% of the sample (62.5%) was considered middle, upper-middle or upper class. In addition, 43.9% of the participants were in a relationship, and the rest were not currently in a romantic relationship. Finally, 34.2% of the sample lived alone, and the rest (65.8%) lived with a partner (38.5%) or relatives (7.5%) or in other conditions (19.8%). Regarding health data, 84.0% of the sample reported having some disease, and 51.9% reported taking medication. In addition, 77% exercised regularly, with aerobic exercise (for example, walking) being the most frequent type. On average, the participants exercised 4.50 days a week for at least 20 minutes (SD = 2.06).

### 3.2 Gold standard scale analysis

An analysis of the internal consistency (Cronbach’s α) of the scales applied was carried out. **Table 3** presents averages, reliability measures for the scales applied and the relationship between the scores using Spearman’s r because the scores did not follow a normal distribution.

**Table 3:** Descriptive results for the gold standard scales.

	GDS-15	GAI	STAXI-2	UCLA
Depression (GDS-15)		.558**	.282**	.450**
Anxiety (GAI)			.415**	.465**
Anger (STAXI-2)				.417**
Loneliness (UCLA)				
No. Items	20	15	10	10
*M*	3.50	6.34	18.87	18.47
*SD*	3.08	5.55	5.84	6.47
Cronbach's α	0.78	0.91	0.84	0.86

GDS-15, Yesavage Geriatric Depression Scale; GAI, Geriatric Anxiety Inventory; STAXI-2, State-Trait Anger Expression Inventory; and UCLA, UCLA Loneliness Scale. **p<.001.

The data obtained reveal solid internal consistency (≥.80) for the anxiety, anger and loneliness scales. As for the depression scale, acceptable internal consistency (≥.70), with a value close to.80, was observed (66). Correlation analysis revealed positive and significant associations between all variables. The loneliness variable had the highest correlations with the other scales.

### 3.3 Dimensionality analysis of the CADIS-G screening instrument

This section presents the results of the EFA, into which the responses to each of the 12 items of the CADIS-G and the scores for the gold standard scales were entered. The CADIS-G items were expected to have significant weight in the factor that corresponded to the respective theoretical scale. That is, items 1–4 were expected to group with the GAI scale (anxiety), items 5–9 were expected to group with the GDS-15 scale (depression), item 10 was expected to group with the STAXI-2 (anger), and items 11 and 12 were expected to group with the UCLA loneliness scale. **Table 4** shows EFA results. According to the KMO value (KMO = 0.844) and the Bartlett test (p <0.001), the data are adequate. In the table, the factorial weights that exceed 0.30 in absolute value have been highlighted.

**Table 4:** EFA of the scales and screening instrument items.

	F1	F2	F3	F4
GDS-15 (DP)	**0.53**	-0.08	0.14	**0.31**
GAI (ANX)	0.06	0.08	0.02	**0.76**
STAXI-2 (ANG)	-0.16	**0.94**	0.15	0.11
UCLA (LON)	**0.34**	0.16	**0.40**	0.17
it. CADIS-G 1 (ANX)	0.02	0.05	0.16	**0.50**
it. CADIS-G 2 (ANX)	-0.11	-0.02	0.10	**0.80**
it. CADIS-G 3 (ANX)	0.05	0.04	-0.14	**0.48**
it. CADIS-G 4 (ANX)	0.15	-0.05	0.05	0.07
it. CADIS-G 5 (DP)	**-0.67**	0.07	0.01	-0.02
it. CADIS-G 6 (DP)	0.28	0.15	0.23	0.03
it. CADIS-G 7 (DP)	**-0.77**	-0.06	0.07	0.05
it. CADIS-G 8 (DP)	**0.48**	-0.03	0.27	0.11
it. CADIS-G 9 (DP)	**-0.59**	-0.08	-0.03	0.05
it. CADIS-G 10 (ANG)	0.26	**0.35**	-0.10	0.09
it. CADIS-G 11 (LON)	0.02	0.01	**0.72**	-0.01
it. CADIS-G 12 (LON)	0.03	0.03	**0.85**	0.03
KMO	0.844			
Bartlett's test	1006.141 (120); *p* <.001			
% explained variance	34.6	9.4	8.5	6.8
Latent factor correlations				
F1: DP	1.00	0.24	0.39	0.50
F2: IRA		1.00	0.14	0.38
F3: SOL			1.00	0.38
F4: ANS				1.00

The factorial weights that exceed 0.30 in absolute value have been highlighted in bold. GDS-15, Yesavage Geriatric Depression Scale; GAI, Geriatric Anxiety Inventory; STAXI-2, State-Trait Anger Expression Inventory; UCLA, UCLA loneliness scale; DP, Depression; ANX, Anxiety; ANG, Anger; LON, Loneliness.

The first factor, depression (DP), was mainly composed of the GDS-15 scale and the CADIS-G items related to depression (items 5, 7, 8 and 9). Additionally, the UCLA loneliness scale presented moderate weight (.34) in this factor, although less than the items of the screening instrument. The second factor (ANG) was composed of anger scores, which included the STAXI-2 scale and item 10 of the CADIS-G. The third factor represented the loneliness variable (LON) because it was mainly composed of items 11 and 12 of the CADIS-G and the UCLA Loneliness scale. Finally, the fourth factor was defined as anxiety (ANX) because the highest weights corresponded to the GAI scale and to the anxiety items of the CADIS-G (items 1, 2 and 3). In this fourth factor, the GDS-15 depression scale presented a moderate weight (.31), although less than the items of the screening instrument. The correlations between the factor scores followed the pattern observed with the gold standards (see **Table 3**). In conclusion, the identified factors partially fit the hypothesis. Regarding the screening instrument, the results suggest that item 4 “I have tried to avoid situations that cause me tension or anxiety” and item 6 “I have given up many activities” should be eliminated because they do not reach factorial loads of.30 for any of the factors.

### 3.4 CADIS-G descriptive

The final version of the CADIS-G screening instrument consists of 10 items (see **Table 1**): 1, 2 and 3 measure anxiety, 5, 7, 8 and 9 measure depression, 10 to measure anger, and 11 and 12 measure loneliness. In the scoring of the depression scale, items 5 (“I have felt satisfied with my life”), 7 (“I have been in good spirits”) and 9 (“I have felt full of energy”) are considered to be inverse; therefore, their scores must be reversed. **Table 5** presents the descriptive data of the instrument for the final 10 items and the scores for the psychological factors.

**Table 5:** Descriptive items of the screening instrument and the final scores.

Item CADIS-G	it. 1	it. 2	it. 3	it. 5	it. 7	it. 8	it. 9	it. 10	it. 11	it. 12
it. 1		.46^**^	.31^**^	-.14^*^	-.27^**^	.28^**^	-.33^**^	.16^*^	.24^**^	.35^**^
it. 2			.34^**^	-.20^**^	-.25^**^	.30^**^	-.14^*^	.23^**^	.25^**^	.36^**^
it. 3				-0.14	-.18^*^	.22^**^	-0.13	.20^**^	.16^*^	0.05
it. 5					.47^**^	-.35^**^	.36^**^	-.20^**^	-0.14	-.16^*^
it. 7						-.35^**^	.47^**^	-.28^**^	-0.12	-.19^*^
it. 8							-.27^**^	.26^**^	.37^**^	.46^**^
it. 9								-.21^**^	-0.12	-.23^**^
it. 10									0.11	0.12
it. 11										.64^**^
it. 12										
*M*	0.98	1.08	0.61	2.25	2.35	0.68	1.97	0.65	0.73	0.54
*SD*	0.96	1.01	0.89	1.00	0.94	1.03	0.98	0.73	1.03	0.90
Total Pt.	Anxiety (it. 1-3)			Depression (it. 5, 7-9)				Anger (it. 10)	Loneliness (it. 11-12)	
*M*	2.68			3.12				0.65	1.27	
*SD*	2.19			2.95				0.73	1.73	
rank	0-9			0-12				0-3	0-6	
α	0.651			0.736				---	0.758	

*p<.050; **p <.001.

The correlations (Spearman’s correlation coefficient) between the items were moderate and, in general, higher between the items that measure the same dimension than with the other items. Regarding the summary scores for the CADIS-G, the reliability indices were good, considering the reduced number of items (α>.65). The correlations (Spearman’s correlation coefficient) between the aggregated scores were moderate. Specifically, the anxiety score showed a correlation of.37 (p <.001) with the depression and anxiety scales and of.27 (p <.001) with the loneliness score. The depression scale showed a correlation of.34 (p <.001) with the anger score and of.31 (p <.001) with the loneliness score. The correlation between loneliness and anger did not reach statistical significance, with a value of.13 (p =.085).

### 3.5 Sensitivity and specificity of CADIS-G

Finally, the ability of the CADIS-G to differentiate people with high levels of anxiety, depression, anger and loneliness was explored using the cut-off points of the gold-standard scales [GAI (anxiety), GDS-15 (depression), STAXI-2 (ANG) and UCLA (loneliness)] as criteria. In our sample, 29.0% (n = 54) of the participants had scores greater than or equal to 10 on the GAI, indicating elevated levels of anxiety; 8.6% (n = 16) of the participants had scores at or above 9 on the GDS-15; and 7.5% of the sample (n = 14) had scores greater than 29 on the STAXI-2. Finally, 13 participants (7%) had scores that are considered indicative of a high degree of loneliness according to the UCLA scale.

Analyzing the results for each dimension of the screening instrument, for the anxiety dimension, the cutoff point was 4, the sensitivity was 0.72, and the specificity was 0.87 (Youden index = 0.60; AUC = 0.85). For the depression dimension, the cutoff point was 6, the sensitivity was 0.81, and the specificity was 0.89 (Youden index = 0.70; AUC = 0.92). For the anger dimension, the cutoff point was 2, the sensitivity was 0.43, and the specificity was 0.92 (Youden index = 0.35; AUC = 0.72). Finally, for the loneliness dimension, the cutoff point was 3, the sensitivity was 0.77, and the specificity was 0.82 (Youden index = 0.59; AUC = 0.84). The CADIS-G results were satisfactory for the anxiety, depression and loneliness dimensions (AUC>.80). However, the anger dimension had lower diagnostic values. This may be due to various causes or a combination of causes: (a) the lack of clear criteria in the STAXI-2 standards to establish a cutoff point, and (b) the use of a single item to measure the anger trait. When considering the trends for this item, scores lower than 2 reflect high levels of specificity, but scores higher than 2 require a more exhaustive evaluation to determine if the individual presents high levels of anger because the sensitivity is only 43%.

## 4 Discussion

The objective of this study was to develop and validate a psychological screening instrument to evaluate anxiety, depression, anger and loneliness in older populations (CADIS-G). During the validation process, two items were eliminated from those initially proposed, resulting in a final version of the CADIS-G with 10 items (3 for anxiety, 4 for depression, 1 for anger and 2 for loneliness). The validation test results indicated that the final version of the CADIS-G successfully measured these emotions, making an important contribution in regard to quickly screening for affective problems in older people. This instrument fulfills a need demanded from sectors such as primary care, a key context for the early detection of affective problems in this population (24, 67).

Another reason for choosing this short tool is to prevent fatigue and incomplete questionnaires, which is especially relevant for the older population. In this demographic, there is a greater risk of subjects leaving questions unanswered, and there may also experience difficulties in interpreting some items (68). Therefore, the development of this screening instrument for elderly individuals helps fill a gap, as many instruments designed for the general population have been used to evaluate older individuals. For example, the STAXI-2 was used as the gold standard for the evaluation of anger (51). When analyzing STAXI-2 scores and the scales, the available options included populations aged16 -19 years, 20-29 years, and 30 years or older. Using these scales assumes that the scores for a 30-year-old subject are more similar to those for a 65-year-old subject than a 25-year-old. It is necessary to specifically address the evaluation of older populations to address these challenges.

Regarding the latter, some of the instruments used in this study as gold standards were designed for older individuals (GDS and GAI), but both have a dichotomous response format (yes/no; agree/disagree). In contrast, the CADIS-G scoring is based on a Likert-type scale with 4 response options, as recommended in validation studies of the GAI (56). Generally, changing the response format in that way improves the psychometric characteristics and properties of any scale or test (69).

The initial proposal for the CADIS-G included 12 items, but items 4 (“I have tried to avoid situations that cause me tension or anxiety”) and 6 (“I have given up many activities”) were eliminated due to inadequate factor loadings for any of the factors. These items were intended to measure the motor aspects of anxiety (avoidance) and depression (loss of activities), but both were unclear and raised doubts for older participants during responses. Other studies on older populations have shown that it is beneficial to formulate items very specifically to avoid generating ambiguity and interpretation issues (70). Therefore, it is necessary to reorganize the CADIS-G item to evaluate these behavioral aspects. The included items presented adequate factor loadings and appropriate levels of reliability for each of the four factors.

Regarding the four CADIS-G items that measure depression, they align with those selected in other studies that validated brief versions of this instrument (32, 67, 71). Of the three items selected to measure anxiety, two were reformulated from the original GAI items (items 1 and 2) and coincide with those selected in another study of an abbreviated version of this instrument (72). In that study, there was comorbidity between anxiety and depression, leading divergent validity problems. In our study, for the anxiety factor, in addition to the high factor loadings for the GAI and the three anxiety items, also showed moderate factor loading for the GDS (0.31). This may related to the high comorbidity between anxiety and depression, which is common in both the general and older population (11). When developing interventions for this population, it is difficult to work with mutually exclusive dimensions, as shown by the high correlations between the scores for the gold standard scales. This comorbidity is also evident in the relationships between depression and loneliness (73), anxiety and loneliness (74), and anger with all of these conditions (75, 76). This highlights the additional challenges faced by this population. The presence of comorbidity is linked to the chronicity of these emotional states (77). For these reasons, we believe that screenings like the one presented in the study are essential for preventing the onset of emotional problems in older adults.

The results allowed us to establish satisfactory cutoff points for the anxiety, depression and loneliness dimensions, optimizing the use of this instrument for identifying possible problems in these areas. The anger dimension showed low sensitivity values. In future versions of the instrument, it would be valuable to include more items related to this emotion. The decision to include only one item was made to prioritize the brevity of the instrument, but it would be interesting to measure other aspects of anger, such as expression and control, for a more comprehensive evaluation. Notably, this type of questionnaire cannot serve as a diagnostic test but rather as a rapid screening method to help classify subjects and identify individuals who may require an in-depth evaluation by mental health professionals. As recommended by Pachana et al. (54), the main considerations for screening tools for older populations should be clarity about the detection purpose, the use of the most appropriate tools, and the ability to manage, interpret and act based on the results. We designed the CADIS-G as an adequate screening tool for older individuals without cognitive impairment, to be used by primary care physicians for the rapid detection of problems related to anxiety, depression, anger, and loneliness. This early identification strategy should be a priority for the care of older adults because various studies have reported that better emotional management is a significant factor in maintaining well-being and quality of life in old age (78, 79) and may have consequences for health and longevity (12). However, there are various barriers for both professionals and older adults that result in a low rate of referral to psychological treatment services (10). Physicians and researchers need screening instruments for this population to improve the care they receive (49). Such tools would help detect potencial emotional disorders more quickly, thus improving their progression and prognosis, and reducing healthcare systems costs (80). In this context, it is noteworthy that an increasing number of screening tools are currently being developed to detect cognitive decline in older adults, leveraging technological advances and artificial intelligence (81–83). Exploring the application of CADIS-G with assessment tools based on this technology could be useful for enhancing its accessibility and reach.

This study has limitations. The sample consists of a population without a mental disorder diagnosis; it would be valuable to test this instrument in a clinical population to assess its diagnostic precision. As mentioned, the evaluation of anger has limitations regarding its sensitivity. In future versions of the CADIS-G, other aspects of anger should be measured, and the number of items should be increased. Additionally, other anger instruments besides the STAXI should serve as the gold standards. Furthermore, the wording of the discarded items should be reviewed to include valid items addressing the motor aspects of anxiety and depression. It is also essential to conduct studies supporting the instrument’s applicability in other languages and cultural contexts. Finally, future studies should explore sociodemographic variables, such as age or whether individuals live alone or with others, institutionalized or not, to analyze if these variables influence the emotional state of older adults.

In conclusion the CADIS-G is a screening instrument with adequate psychometric properties to measure anxiety, depression, anger and loneliness in older individuals. The subscales of the instrument are correlated with the scores of gold standard instruments used for validation. Additionally, the reliability of the CADIS-G subscales is acceptable after reducing the number of items. Regarding the CADIS-G ability to identify individuals with high levels of these variables, the results show good sensitivity and specificity for three of the four dimensions, with sensitivity for anger being improvable. The CADIS-G could be a useful instrument in contexts such as primary care, where, due to system overload, in-depth examinations of patients’ psychological states may not be feasible. Policymakers and healthcare professionals should prioritize integrating these cost-effective interventions into senior care to improve limited access to care and the long-term impact of interventions (84). The CADIS-G could facilitate a rapid assessment (approximately 5 minutes). The instrument could be integrated into the interview protocols of doctors, nurses, social workers, and others, serving as an initial indicator and enabling more in-depth evaluations of the psychological state of older individuals, if necessary.

## Data Availability

The raw data supporting the conclusions of this article will be made available by the authors, without undue reservation.
